# Bioactive peptides PDBSN improve mitochondrial function and suppression the oxidative stress in human adiposity cells

**DOI:** 10.1080/21623945.2023.2278213

**Published:** 2023-11-09

**Authors:** Huiping Shen, Yong Lei, Wen Xie, Tieliang Ma, Li Bao, Qin Gao, Bingyu Chen, Biao Dai, Dani Qin

**Affiliations:** Department of Pediatrics, Yixing People’s Hospital, Yixing, Jiangsu, China

**Keywords:** Adipocyte, obesity, PDBSN, mitochondrial, ROS

## Abstract

Mitochondria are essential for generating cellular energy and are significant in the pathogenesis of obesity. Human visceral and subcutaneous preadipocytes (HPA-v and HPA-s) were cultured into mature adipocytes. Intracellular triglyceride (TG) content was assessed using oil-red O staining and tissue triglyceride determination. Mitochondrial membrane potential (MMP) and reactive oxygen species (ROS) levels were measured with fluorescent indicators. Gene and protein expression related to mitochondrial biogenesis were analyzed by real-time quantitative PCR and Western blotting. Morphological changes were observed via electron microscopy. Results show that PDBSN significantly increased MMP while decreasing TG and ROS levels. The transcription and protein levels of PGC1-α and MTFA were upregulated, and mitochondrial fusion and fission markers (MFN1, MFN2, NRF1, DRP1) were elevated. Additionally, PDBSN enhanced maximum respiratory capacity and reduced ROS. These findings suggest that PDBSN improves mitochondrial function, providing insights for obesity treatment and metabolic disease management.

## 1. Introduction

Obesity is one of the strongest risk factors for immune disorders, insulin resistance, type 2 diabetes mellitus (T2DM), cardiovascular disease (CVD), and some cancers [1–4]. This causes both a decline in quality of life and life expectancy, and obesity has become one of the most serious health problems in modern society. At present, prevention and treatment strategies for obesity mainly focus on lifestyle and behavioural interventions to reduce energy intake and increase energy expenditure, but their effects are limited [5]. Surgical treatment is only applicable to patients with severe obesity and safety risks [6]. Therefore, it is necessary to develop safer methods to prevent and treat obesity effectively.

Given the recent development of polypeptide omics, polypeptides in obesity and metabolic-related diseases have been shown as favourable prospects for the effective treatment of obesity. Several reports have demonstrated the roles of peptides such as leptin [7], neuropeptide Y, and peptide YY [8] in the treatment of obesity. In previous studies, PDBSN reduced lipid accumulation in adipocytes and ameliorated high-fat diet (HFD)-induced obesity and metabolic homoeostasis [9,10]. Peptides have become a novel research topic for the treatment of metabolic diseases, such as obesity and diabetes [11]. Mitochondria serve as the pivotal organelles responsible for energy metabolism in adipocytes, and their malfunction is intricately associated with the onset of metabolic disorders and obesity-related ailments [12]. Notably, investigations have substantiated that enhancing mitochondrial functionality holds promise in averting metabolic diseases [13]. Further studies are required to determine the relationship between PDBSN, mitochondrial function, and energy metabolism.

Therefore, in vitro studies were performed to investigate the effects of PDBSN on mitochondrial DNA (mtDNA) copy number, morphology, biogenesis, ROS production, and mitochondrial membrane potential (MMP) in adipocytes. Moreover, we detected PDBSN-mediated modification of mitochondrial respiration. PDBSN may serve as a pharmacological agent to enhance mitochondrial function, thereby establishing a theoretical foundation and potential therapeutic target for the prevention and treatment of obesity.

## 2. Results

### 2.1. Localization and the cytotoxicity of PDBSN in pre-adipocytes

**Figure 1.**
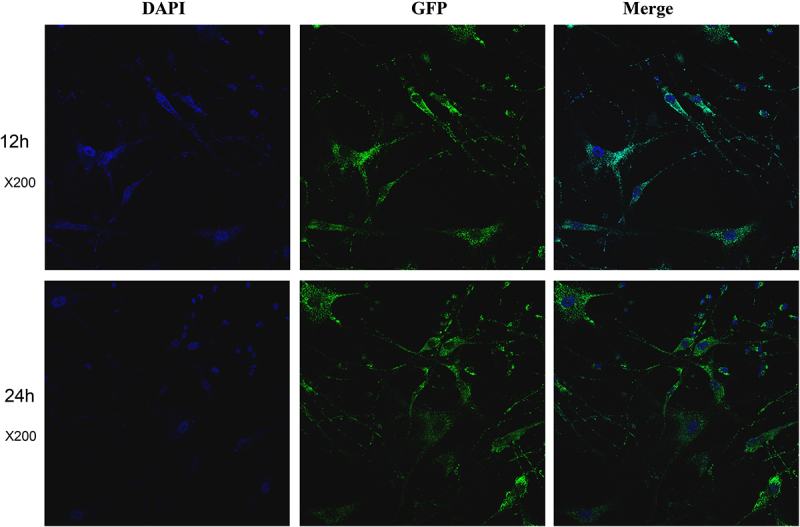
Localization and the cytotoxicity of PDBSN in pre-adipocytes. HPA-v cells were selected and exposed to 20 μM FITC-labelled PDBSN and observed by laser scanning confocal microscopy at 12 and 24 h.

HPA and HPA-s cells were exposed to 20 μM FITC-labelled PDBSN and observed using laser scanning confocal microscopy. The results showed that most cells exhibited obvious fluorescence (Figure 1 and supplemental Fig. S1), indicating that PDBSN could cross the cell membrane and localize within the cytoplasm or even the nucleus, suggesting that PDBSN possesses good cell-penetrating activity.

### 2.2. PDBSN significantly reduced intracellular levels of TG

**Figure 2.**
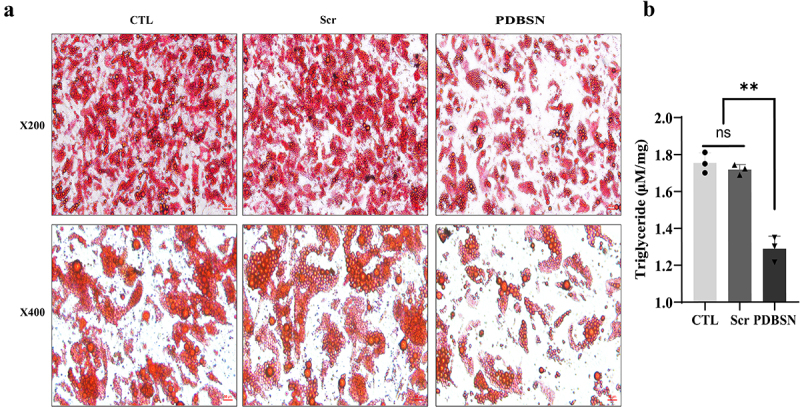
PDBSN significantly reduced intracellular levels of lipids. the same dose of Scr or PDBSN was administered during the differentiation of HPA cells. (a) oil red staining. Cells were subjected to oil red O staining on day 7 and analyzed by optical microscopy at 200× and 400× magnification. (b) effect of PDBSN on intracellular TG. The intervention was performed in the differentiation process of HPA cells, Scr was given under control, the experimental group was given the same dose of PDBSN, and the intracellular TG level was measured on day 7 of cell differentiation and maturation. Values represented the means ± SD from three independent experiments. *p < 0.05.

Lipid deposition in adipocytes plays an important role in mitochondrial dysfunction. The results obtained from oil red O staining and triglyceride kit analysis revealed a significant reduction in intracellular TG levels and oil red staining, indicating a substantial decrease in lipid deposition within the PDBSN group. This indirectly demonstrated that PDBSN could reduce the accumulation of lipids, as lipids are stored as TGs within the cells (Figure 2 and supplemental Fig. S2).

### 2.3. PDBSN reduced intracellular ROS levels, increased MMP and ATP

**Figure 3.**
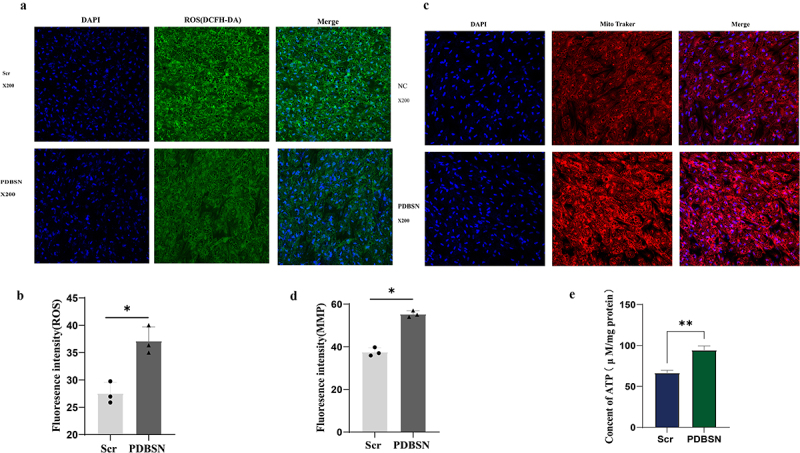
Effects of PDBSN on ROS levels, MMP and ATP. after differentiation, the adipocytes were treated with the same dose of Scr or PDBSN for 24 h. (a) mitochondrial membrane potential was visualized using a Mito Tracker red fluorescence probe. (b) the mitochondrial membrane potential in adipocytes was visualized by fluorescence microscopy. (c) Total ROS levels were measured by flow cytometry using a DCF fluorescence probe and ROS levels in cardiomyocytes were observed by fluorescence microscopy (d). (e) intracellular ATP content was determined by Luminescent ATP detection Assay, according to the manufacturer’s instructions. Values represented are the means ± SD from three independent experiments. **p < 0.001.

Excessive ROS production plays an important role in mitochondrial dysfunction. Mitochondria, as the main site of energy metabolism, are closely related to MMP. We examined the impact of PDBSN on MMP to assess its effect on mitochondrial function. Additionally, intracellular ROS levels, which are closely related to mitochondrial dysfunction, significantly decreased (Figure 3a). As expected, MMP and ATP contents in adipocytes were significantly higher than those in the control group (Figure 3b,c). These results indicated that PDBSN can significantly reduce intracellular ROS, increase MMP, and promote ATP production, suggesting that PDBSN is closely related to mitochondrial function.

### 2.4. PDBSN promoted mitochondrial biosynthesis

**Figure 4.**
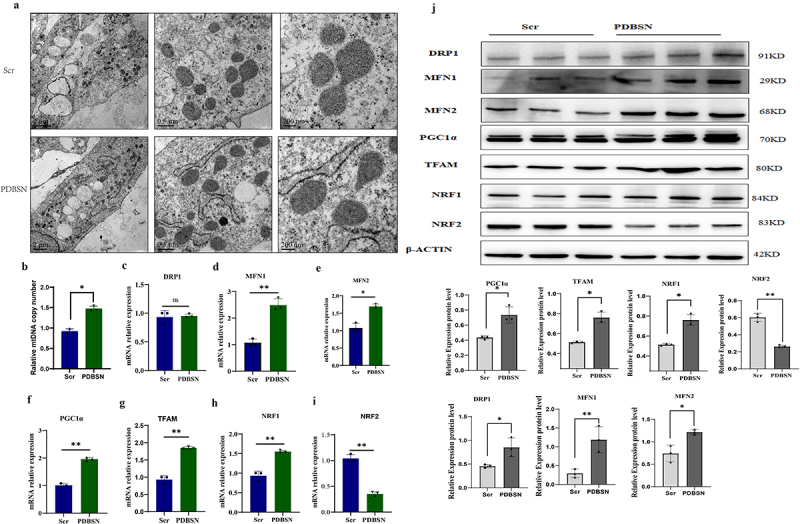
Effects of PDBSN on mitochondrial number and biosynthesis. the same dose of Scr or PDBSN was administered during the differentiation of HPA cells. (a) Ultrastructure of the mitochondria was visualized by transmission electron microscopy. (b) Total DNA was extracted and relative expression levels of mtDNA copy number (ND1) were determined by real-time qPCR and 28 S was used to normalize results. (c-i) RNA was extracted and relative mRNA expressions of PGC1α, TFAM, NRF1, NRF2, DRP1, MFN1, and MFN2 were examined by real-time qPCR. PPIA mRNA was used as an internal control. Values are shown as mean ± SD of triplicate experiments. (j) Total protein was isolated from stable cell lines and analysed by western blot using antibodies against PGC1α, TFAM, NRF1, NRF2, DRP1, MFN1, and MFN2. (k) β-actin protein was used as an internal control. Values represented are the means ± SD from 3three independent experiments. *p<0.05, **p<0.001

PGC1α and MTFA are the main proteins involved in mitochondrial biogenesis. Their expression significantly increased in adipocytes after PDBSN treatment (Figure 4a). The electron microscopy results revealed a high abundance of mitochondria, endoplasmic reticulum, and lipid droplets of varying sizes within the cytoplasm of both visceral and subcutaneous adipocytes. The PDSBSN group exhibited significantly higher mitochondrial count and reduced incidence of mitochondrial swelling and vacuolation in both types of adipocytes than those of the control group. Furthermore, subcutaneous fat cells displayed a significantly greater number of mitochondria compared to visceral fat cells (Figure 4b and supplemental Fig. S4). To demonstrate the role of PDBSN in regulating mitochondrial number, mitochondrial number and MMP were measured. RT-qPCR results showed that mitochondrial DNA (mtDNA) levels increased by approximately 30% in PDBSN-treated adipocytes (Figure 4c). Adipocytes exhibit varying sizes and irregular shapes, including long spindle or oval forms. Mitochondria are stained red using Mito Tracker, displaying high fluorescence content with an uneven distribution. The perinuclear region demonstrates intense fluorescence given the concentrated presence of mitochondria, and mitochondrial fluorescence is also observed in the cytoplasm. Notably, both perinuclear and cytoplasmic mitochondrial fluorescence levels are higher compared to control cells (Figure 4d). The results of laser confocal microscopy staining with the mitochondrial probe Mito Tracker™ Red showed that the red fluorescence intensity in the PDBSN group increased by approximately 30%. We further examined the expressions of *MFN1*, *MFN2*, *NRF1*, and *DRP1*, which mediate mitochondrial fusion and fission (Figure 4e). These results suggest that PDBSN promotes mitochondrial fusion and fission. Although these results indicated that PDBSN can promote mitochondrial biogenesis to some extent, it is important to note that mitochondrial synthesis is in dynamic equilibrium. Therefore, fully understanding the role of PDBSN in mitochondrial biogenesis based on these data may be challenging.

### 2.5. PDBSN increased mitochondrial respiration

**Figure 5.**
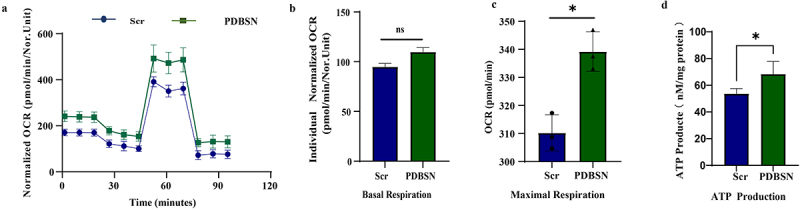
Effects of PDBSN on mitochondrial respiratory function. after differentiation, the adipocytes were treated at the same dose of Scr or PDBSN for 24 h. Mitochondrial respiratory function, analysed using the Seahorse XF mitochondrial stress test analyser, was significantly increased in adipocytes following PDBSN administration (Figure 5a). Seahorse profile for OCRs of the two groups treated with 1.5 μM oligomycin, 0.5 μM FCCP, and 1.5 μM antimycin/rotenone. (b) Basal/maximal respiratory capacity, (c) maximal respiratory capacity, and adenosine triphosphate (ATP) production. Values represent the mean ± SD from three independent experiments. *p<0.05

Mitochondrial respiratory function, including basal/maximal respiratory capacity and ATP production, as analysed using the Seahorse XF Mitochondrial Stress Test analyser, was significantly increased in PDBSN-treated adipocytes (Figure 5 and supplemental Fig. S5). These results suggest that PDBSN can improve mitochondrial function by enhancing mitochondrial maximal respiration capacity and promoting ATP production both in HPA-v and HPA-s cells.

## 3. Discussion

Mitochondria are the energy centres of adipocytes and are involved in many key metabolic functions, such as adenosine triphosphate (ATP) production, fatty acid synthesis and oxidation, and cellular triglyceride balance [12,14,15]. Owing to the important role of mitochondria in energy metabolism, mitochondrial dysfunction plays a key regulatory role in the pathophysiology of obesity and diabetes [16]. Mitochondrial dysfunction of white adipose tissue in obesity has long-term effects on adipose tissue and systemic metabolism [12]. Downregulation of mitochondrial function or biogenesis in white adipose tissue is a major driver of obesity-related metabolic diseases [17]. Elucidating the pathways involved in mitochondrial dysfunction could help identify targets for drugs or interventions to enhance mitochondrial biogenesis or function in adipose tissue [17]. In the present study, we concluded that PDBSN could reduce cellular ROS levels, promote mitochondrial biosynthesis, and even increase mitochondrial respiration.

Adipocytes are dynamic reservoirs for energy storage and consumption. The mitochondria play a central role in energy metabolism by converting nutrients into energy. Most notably, they are responsible for producing the vast majority of ATP during cellular respiration, which is the conversion of chemical energy from nutrient substrates to ATP via oxidation [18]. Excessive energy accumulation leads to fat accumulation and is related to metabolic diseases such as metabolic syndrome and insulin resistance [18]. Low aerobic capacity [19], and low resting metabolic rate are associated with weight gain [20]. In the current study, we found that PDBSN improved MMP and ATP production. Consequently, PDBSN may regulate energy expenditure by increasing the maximum mitochondrial respiration and ATP production in adipocytes.

To investigate the effects of PDBSN on mitochondrial function-related indicators, we investigated mitochondrial ROS levels. Mitochondria are the main source of ROS, and the imbalance between ROS and cellular stress defence mechanisms leads to a vicious cycle of further mitochondrial dysfunction, resulting in increased ROS, which in turn promotes more damage and energy imbalance, eventually leading to energy metabolism-related diseases, such as obesity and insulin resistance [18]. ROS production under these conditions may further aggravate the mitochondrial structural and functional damage. In addition, the production of ROS from the electron transport chain and other sources may play a positive role in insulin signalling and enhance insulin sensitivity [21]. It has been proposed that the accumulation of mitochondrial dysfunction, including excessive ROS production and reduced fat oxidation capacity, plays a major role in this process [22]. Previous studies have demonstrated that peptides from dietary sources, like RDPEER isolated from watermelon seeds, can inhibit oxidative stress by reducing ROS production [23]. In this study, ROS levels were significantly reduced after incubation with PDBSN, suggesting that PDBSN plays a role in maintaining the redox balance.

Impairment of mitochondrial function reduces mitochondrial biogenesis and mitochondrial DNA content [17]. Mitochondria are structurally dynamic and can change their shape, size, and number through fusion and fission processes [18]. Mitochondrial biogenesis is achieved by induction or post-translational modifications such as PGC1α [24], NRF-1/2 [24], or mitochondrial transcription factor A (TFAM) [25]. PGC1α is regulated by transcriptional and post-translational modifications, including AMPK phosphorylation (activated by high AMP/ATP ratios) [26]. In a previous study, PDBSN activated the AMPK pathway, suggesting that PGC1α might be regulated by phosphorylation by AMPK. Once activated by phosphorylation or deacetylation, PGC1α activates NRF1 and NRF2, which in turn activates the mitochondrial transcription factor (TFAM) [27]. TFAM has been cloned as a transcription factor for mtDNA and is essential for mtDNA maintenance [24]. Activation of the PGC1α-NRF-TFAM pathway leads to increased mitochondrial DNA and protein synthesis, which in turn promotes the generation of new mitochondria [27]. Nrf2 is an important leucine zipper transcription factor that controls the expression of a variety of antioxidant proteins in response to ROS stress and mitochondrial biogenesis [28,29]. Extracellular redox status regulates Nrf2 activation through mitochondrial reactive oxygen species [30]. In this study, we found that Nrf2 expression was reduced at both the transcript and protein levels. Regarding the inhibitors of Nrf2, one report proposed that BRD4 (bromodomain-containing protein 4) is a transcription cofactor that has recently been identified as an inhibitor of the Nrf2 pathway [31]. However, another report showed that GSK3β, which is known to inhibit Nrf2 activity in a βTrCP-proteasome-dependent manner, is activated in diabetic foetal endothelial cells [32]. Further studies are required to clarify this issue.

Mitochondrial fusion is mainly controlled by Mfn1 and Mfn2, whereas mitochondrial fission is mainly regulated by Drp1 [16]. Single knockout of Mfn1 or Mfn2 led to mitochondrial disruption in mouse embryonic fibroblasts (MEF) [33], and double knockout of Mfn1 and Mfn2 completely blocked mitochondrial fusion [34,35]. Conversely, mitochondrial fusion can be rescued by overexpression of Mfn1 or Mfn2 [33]. These findings suggest that Mfn1 and Mfn2 play important roles in mitochondrial fusion. Drp1 is involved in the development of obesity and T2D through its effect on mitochondrial oxidative metabolic rate [36]. In this study, we found that after PDBSN intervention, Mfn1 and Mfn2 levels improved while the Drp1 level was unchanged. However, further studies are needed to clarify the different regulatory mechanisms of Mfn1, Mfn2, and Drp1 targeting mitochondrial metabolism in diabetes and obesity.

In summary, cellular-level studies have provided preliminary evidence supporting the promotion of mitochondrial production and improvement in mitochondrial aerobic respiration capacity by PDBSN; however, further investigations at the animal level are warranted. The availability of peptides in animal studies requires consideration of several factors, including peptide stability, solubility, and membrane permeability. Therefore, by refining the peptide’s structure and properties and advancing drug release techniques and in vivo efficacy evaluation methods, we expect to conduct in-depth animal-level research on PDSBSN. Despite these limitations, we believe that our study provides important new insights into the function of mitochondria in obesity.

## 4. Materials and methods

### 4.1. Cell culture and reagents

HPA-v cells (Cat. No.7210, Carlsbad, CA, USA) derived from human visceral adipose tissue were obtained from ScienCell Research Laboratories [37]. HPA-v cells were cultured according to our previous experimental protocol [38]. During the differentiation of adipocytes, the cells in the experimental group were treated with 20 μM PDBSN for 1, 3, and 5 days, and an equal volume of scramble peptide solution was used as the control. The PDBSN peptide was synthesized by Shanghai Ketide Biotechnology Co. Ltd. Its N-terminus was linked to a vector peptide (GRKKRRQRRRPPQQ) derived from the hivat sequence. The scrambled peptide (Scr) was used as a control (GRKKRRQRRRPPQQ-MNAVSLELADLGSKI) at a concentration of 20 μM. To detect peptide localization, PDBSN was used to link fluorescein isothiocyanate to the N-terminal fluorescein isothiocyanate (FITC) (FITC-GRKKRRQRRRPPQQGLSVADLAESIMKNL).

### 4.2. Laser scanning confocal microscopy

The HPA-v and HPA-s pre-adipocyte cells were seeded in a 6-well plate, with approximately 1 × 10^5^ cells per well. HPA-v cells were selected and exposed to 20 μM FITC-labelled PDBSN and observed by laser scanning confocal microscopy at 12 and 24 h. The following steps were performed in accordance with the instructions provided by the Hochest 33,258 staining kit (Beyotime, Shanghai, China). The stained cells were imaged using laser scanning confocal microscopy (Leica TCS SP8).

The HPA-s cells were selected and exposed to 20 μM FITC-labelled PDBSN and observed by laser scanning confocal microscopy at 48 h. The following steps were performed in accordance with the instructions provided by the Hochest 33,258 staining kit (Beyotime, Shanghai, China). The stained cells were imaged using the CQ1 dual-rotating confocal laser high-intensity imaging analysis system (Yokogawa).

### 4.3. Oil red O staining

The cells were cultured, fixed, and stained according to our previous experimental methods [38], and finally photographed.

### 4.4. Triglyceride measurements

Intracellular triglyceride content was determined using the Tissue Triglyceride Assay kit (E1013, Applygen, Beijing, China) according to our previous experimental protocol [38].

### 4.5. Real-time quantitative PCR

Total RNA extraction, quantification, and selection of internal controls were performed according to our previous experimental protocol [38]. All primer sequences are listed in Table 1.

**Table 1.** Oligonucleotide sequences for primer/probe sets used in TaqMan analysis.

Gene.	Forward primer (5’-3’)	Reverse primer (5’-3’)	Probe
Cyt B	CAACATCTCCGCATGATGAAA	CCATAATTTACGTCTCGAGTGATGTG	CCATGCACTACTCACCAGACGCCTCAA
−ACTIN	GGCGGCCAAGCGTTCATAG	AGGCGTTCAGTCATAATCCCACAG	
PGC-1α	CGGAAATCATATCCAACCAG	AAAATTGCTTGCGTCCAC	
mtTFA	GGAATGTGGAGCGTGCTAAAA	TGCTGGAAAAACACTTCGGAATA	
β-actin	CCTGAGGCTCTTTTCCAGCC	TAGAGGTCTTTACGGATGTCAACGT	

### 4.6. MtDNA quantification

Mitochondrial DNA (mtDNA) was quantified using real-time PCR Nuclear DNA and mtDNA were compared using quantitative real-time PCR. The mtDNA ratio reflects the cell concentration of mitochondria in a tissue. Therefore, CytB was selected for quantification of mtDNA according to a previously described method [39]. We amplified part of the cyt b mitochondrial gene using specific primers (Table 1) and a specific probe [40]. β-Actin and cyt b were quantified using real-time PCR. Data are expressed as the ratio of mean mitochondrial DNA (cyt b) to mean nuclear DNA (β-actin) (mtDNA/β-actin).

### 4.7. Western blotting

Primary antibodies to MFN1, MFN2, PGC1α, and DRP1 were obtained from Cell Signaling Technologies, Inc. (Danvers, MA, USA). NRF1, NRF2, TFAM, HRP-labelled anti-mouse, and anti-rabbit secondary antibodies were purchased from Proteintech (Chicago, IL, USA). Cell lysis, protein extraction, and image acquisition were performed according to our previous methods [38].

### 4.8. Transmission electron microscopy detection

Adipocytes were then digested with trypsin on day 7, washed in fresh PBS (pH 7.4), and fixed in 2.5% glutaraldehyde and 4% paraformaldehyde in the same buffer. After washing with 0.1 M cacodylate buffer, the cells were fixed with 1.5% potassium ferrocyanide and 1% osmium tetroxide for 1 h, washed with 1X PBS three times, fixed with 1% osmium tetroxide for 1 h, stained with 1% uranyl acetate water for 30 min, and finally dehydrated with a graded series of ethanol to 100%. The samples were infiltrated and embedded in TAAB Epon (Marivac Canada Inc., St. Laurent, Canada). Ultrathin sections (60 nm) were examined using a JEOL JEM-1010 transmission electron microscope at an accelerating voltage of 80 kV.

### 4.9. ATP production

The cells were induced to differentiate, as described above. Adipocytes were incubated with Scr or PDBSN for 24 h. ATP content was determined using a fluorescein-based luminescence detection kit (Beyotime, Beijing, China) using Varioskan LUX (Thermo Scientific, MA, USA).

### 4.10. Measurement of MMP

Adipocytes were incubated with Scr or PDBSN for 24 h, stained with MitoTrackerRed FM (Thermo Fisher Scientific, California, USA), and the mitochondrial count was analysed by flow cytometry. Adipocytes were incubated with MitoTracker at 37°C for 30 min and washed thrice with preheated PBS. Flow cytometry (FACS) was performed using an excitation wavelength of 579 nm and an emission wavelength of 644 nm.

The control group and experimental group were treated with Scr or PDBSN for 24 h respectively, stained with Mito Tracker (0.5 mL/well) for 45 min, and washed with PBS. After fixation with paraformaldehyde, all nuclei were stained with DAPI (4 ’, 6-diamidino-2-phenylindole) (C1002, Beyotime Biotechnology Institute, Nantong, Jiangsu, China). Images were taken using a confocal fluorescence microscope (Leica, Solms, Germany), and the fluorescence intensity reflected MMP.

### 4.11. Measurement of intracellular ROS content

The adipocytes were incubated with Scr or PDBSN for 24 h, then incubated with 5 μM H2-DCFDA (#S0033S, Beyotime Institute of Biotechnology, Shanghai, China) at 37°C for 30 min, then washed with PBS three times, and analysed by FACS (excitation at 488 nm, emission at 525 nm).

After differentiation, the control and experimental groups were treated with Scr and PDBSN for 24 h, stained with H2-DCFDA (0.5 mL/well) for 30 min, and washed with PBS three times. All the nuclei were stained with Hoechst (#C1027, Beyotime Biotechnology Institute, Shanghai, China). Images were taken using a confocal fluorescence microscope (Leica, Solms, Germany), and the fluorescence intensity reflected the ROS.

### 4.12. Seahorse experiments

The mitochondrial respiratory capacity was measured using Seahorse experiments. An Agilent Seahorse Xfe96 Extracellular Flux Analyzer (Agilent, Delaware, USA) and Agilent Seahorse XF Cell Mito Stress Test Kit (Agilent, Delaware, USA) were used to directly measure the oxygen consumption rate (OCR). First, 1 × 10^5^ cells were seeded in each well of 96-well Seahorse XF cell culture microporous plates (Agilent, Delaware, USA) and treated as described above. Seahorse XF calibration solution was then used to hydrate the sensor probe plate overnight in a CO_2_-free incubator at 37°C. Antimycin A, FCCP, and oligomycin A were added to each well and the concentrations were calculated.

### 4.13. Statistical analysis

All data are presented as the mean ± SD of three independent experiments. Echocardiographic parameters were compared between the different treatment groups using a paired t-test. Data were analysed using the Prism 7 software (GraphPad, San Diego, CA, USA). Differences were considered statistically significant at *p* < 0.05.

## Supplementary Material

Supplemental Material

## Data Availability

The data sets used and/or analysed during the current study are available from the corresponding author upon reasonable request.

## Abbreviations

HPA-v, Human primary visceral pre-adipocytes; HPA-s, Human primary subcutaneous pre-adipocytes; TG, Triglyceride; ROS, Reactive oxygen species; MMP, Mitochondrial membrane potential; T2DM, Type 2 diabetes mellitus; CVD, Cardiovascular disease; HFD, High-fat diet; mtDNA, Mitochondrial DNA; Scr, Scrambled peptide; OD, Optical density; PPIA, Peptidylprolyl isomerase A; ATP, Adenosine triphosphate; TFAM, Activates the mitochondrial transcription factor; MEF, Mitochondrial disruption in mouse embryonic fibroblasts.
