# Supplementary figures and images for: Bioactive peptides PDBSN improve mitochondrial function and suppression the oxidative stress in human adiposity cells
Source: Adipocyte. 2023 Nov 9;14(1):2278213. doi: 10.1080/21623945.2023.2278213 (PMC12184117; doi:10.1080/21623945.2023.2278213)

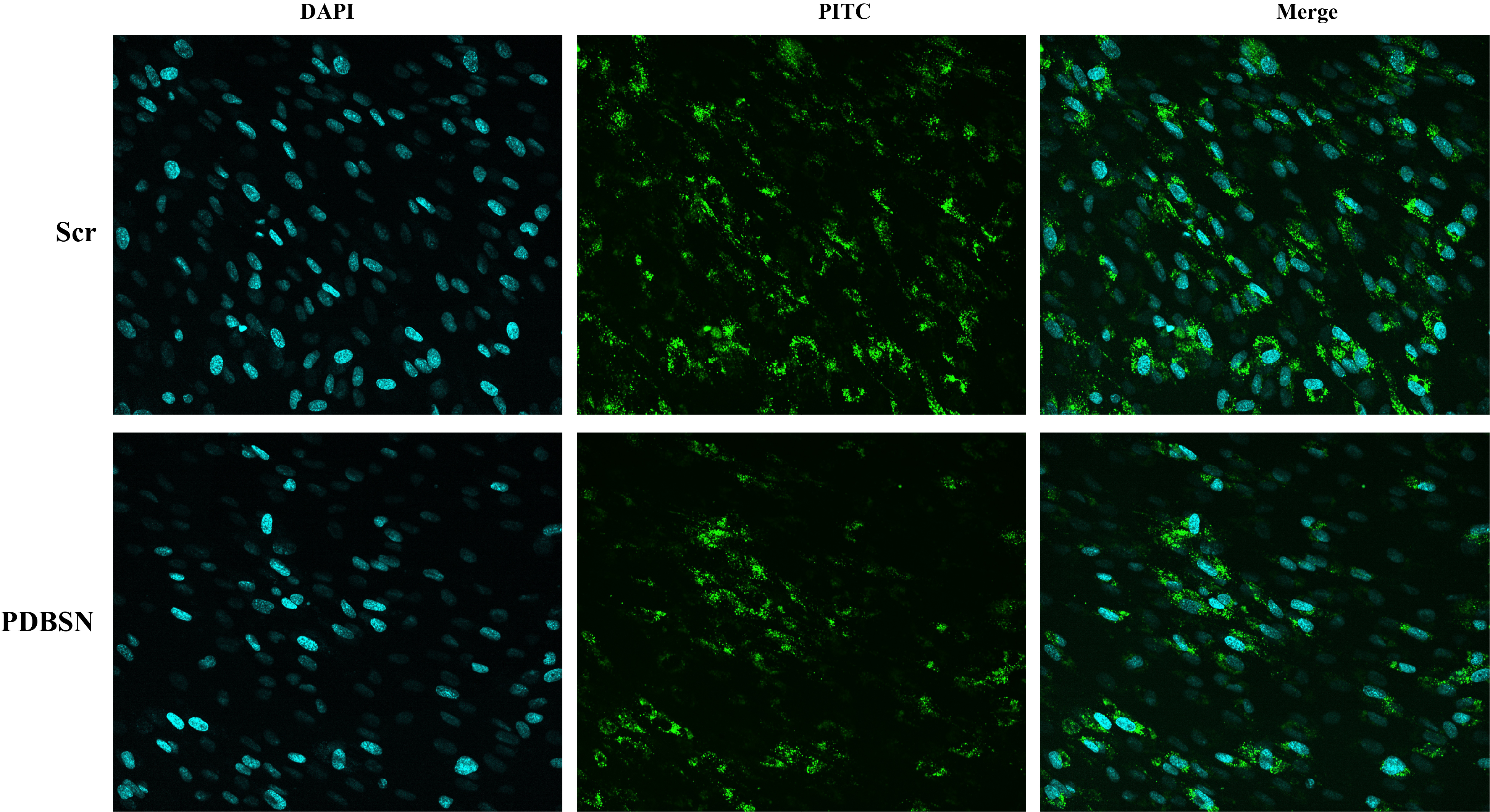

Supplement: Supplemental Material [file KADI_A_2278213_SM1551.zip › supplemental Fig. 1.jpg]

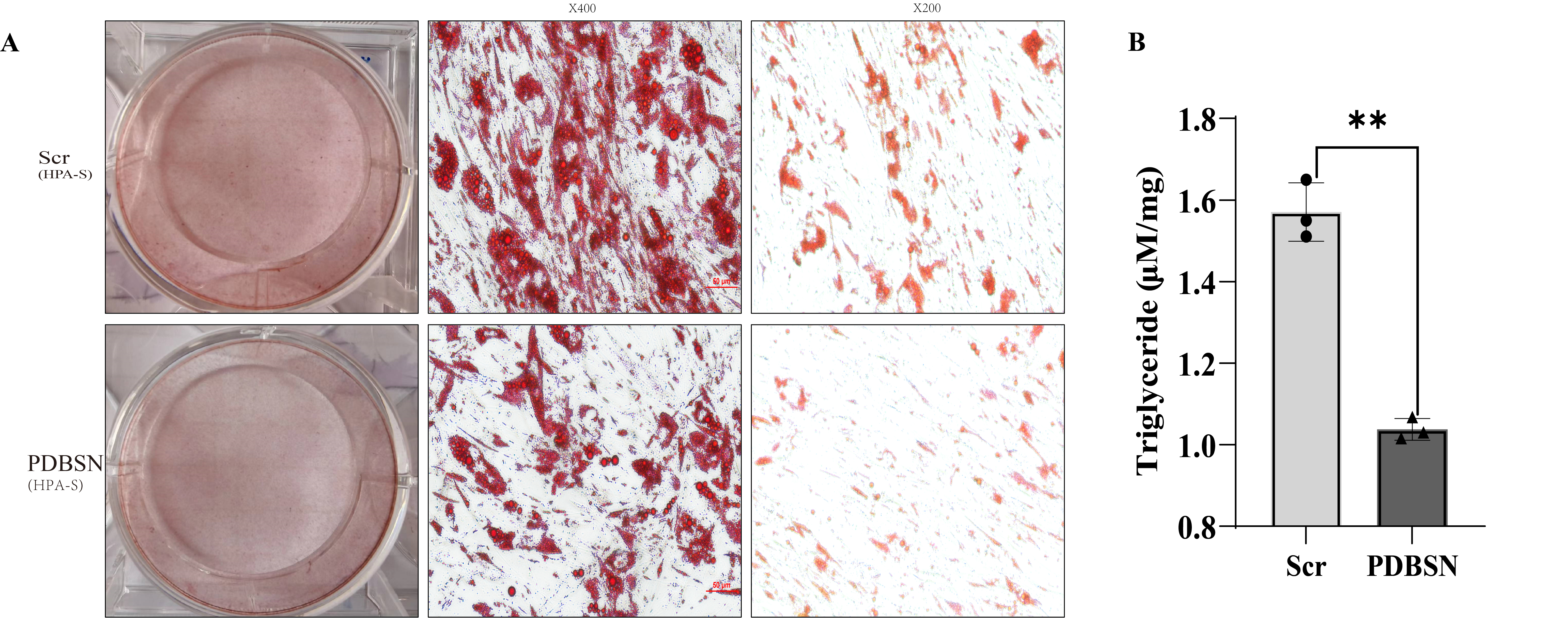

Supplement: Supplemental Material [file KADI_A_2278213_SM1551.zip › supplemental Fig. 2.jpg]

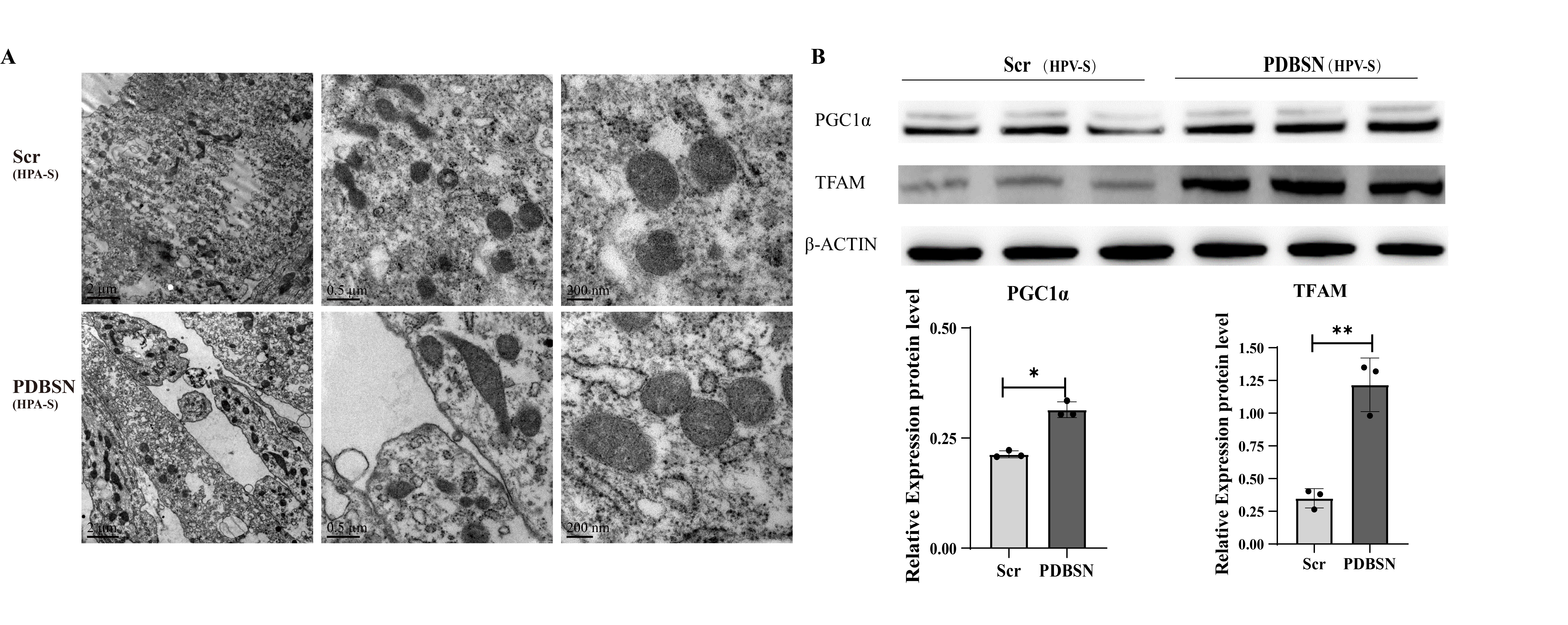

Supplement: Supplemental Material [file KADI_A_2278213_SM1551.zip › supplemental Fig. 4.jpg]

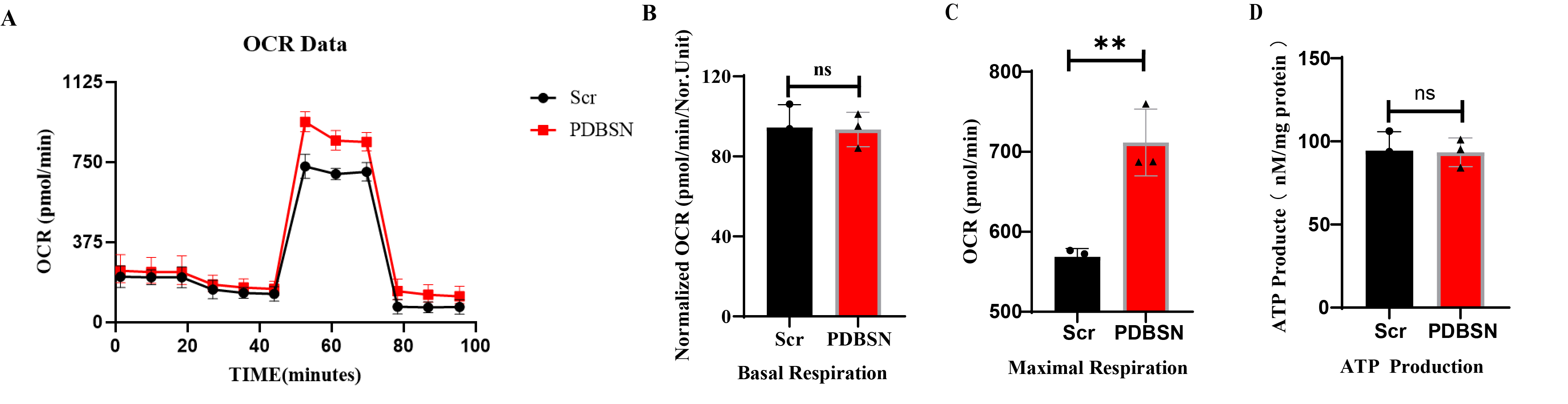

Supplement: Supplemental Material [file KADI_A_2278213_SM1551.zip › supplemental Fig. 5.jpg]
